# Altered mitochondrial DNA copy number contributes to human cancer risk: evidence from an updated meta-analysis

**DOI:** 10.1038/srep35859

**Published:** 2016-10-24

**Authors:** Liwen Hu, Xinyue Yao, Yi Shen

**Affiliations:** 1 Department of Cardiothoracic Surgery, Jinling Hospital, School of Clinical Medicine, Nanjing University, Nanjing, Jiangsu Province, P. R. China; 2 Institute of Laboratory Medicine, Jinling Hospital, School of Clinical Medicine, Nanjing University, Nanjing, Jiangsu Province, P. R. China

## Abstract

Accumulating epidemiological evidence indicates that the quantitative changes in human mitochondrial DNA (mtDNA) copy number could affect the genetic susceptibility of malignancies in a tumor-specific manner, but the results are still elusive. To provide a more precise estimation on the association between mtDNA copy number and risk of diverse malignancies, a meta-analysis was conducted by calculating the pooled odds ratios (OR) and the 95% confidence intervals (95% CI). A total of 36 case-control studies involving 11,847 cases and 15,438 controls were finally included in the meta-analysis. Overall analysis of all studies suggested no significant association between mtDNA content and cancer risk (OR = 1.044, 95% CI = 0.866–1.260, P = 0.651). Subgroup analyses by cancer types showed an obvious positive association between mtDNA content and lymphoma and breast cancer (OR = 1.645, 95% CI = 1.117–2.421, P = 0.012; OR = 1.721, 95% CI = 1.130–2.622, P = 0.011, respectively), and a negative association for hepatic carcinoma. Stratified analyses by other confounding factors also found increased cancer risk in people with drinking addiction. Further analysis using studies of quartiles found that populations with the highest mtDNA content may be under more obvious risk of melanoma and that Western populations were more susceptible than Asians.

Mitochondria are organelles in eukaryotic cells that participate in a wide range of biological actions, such as energy metabolism, free oxygen radical generation, and cell apoptosis^1^. Usually thousands of mitochondria exist in each cell. Mitochondria possess separate copies of DNA (mtDNA), which are double-stranded, maternally inherited circular nucleotides with 16,569 base pairs encoding for 37 RNAs, including 13 mRNA, 22 transfer RNAs and two ribosomal RNAs. mtDNA content in cells typically ranges from 10^3^ to 10^4^ copies per cell and varies by cell type^2^.

Due to the lack of protection from introns and histones, and less efficient DNA repair mechanisms, mtDNA is particularly susceptible to reactive oxidative species (ROS) and other sources of genotoxic damage, which may finally lead to sequence mutations or copy number alterations^3^. Changes in mtDNA copy number could alter the expression of mitochondrial genes and result in abnormal mitochondrial functions, such as energy production, signaling transduction, apoptosis and cell growth. Therefore, aberrant mtDNA content could potentially lead to alterations in oxidative phosphorylation, and enhance the production of ROS in aerobic metabolism, which can cause chromosomal aberrations such as base pair mutation and chromosome rearrangements, and finally facilitate the malignant transformation^4^. Contribution of mtDNA copy number alterations to nuclear genome damage has been confirmed by previous studies. Corral *et al*. found that introduction of mtDNA molecules to chemically-induced rat hepatomas and HT-1080 fibrosarcoma cell line caused an increased number of copies of COI, COII and COIII pseudogenes in the nuclear genome^5^, while Delsite *et al*. demonstrated that a reduction in mitochondria in HeLa cells resulted in increased lipid peroxidation^6^. It has been shown that increased lipid peroxidation could induce damage directly to nuclear DNA^7^.

Previous studies have reported quantitative changes in mtDNA content in patients’ cancerous tissues compared with their normal counterparts, including an increase of mtDNA content in breast cancer and endometrial adenocarcinoma^8^,^9^, and a decrease in colorectal cancer, cervical cancer, osteosarcoma and Ewing’s sarcoma^10^,^11^,^12^,^13^. Moreover, the alteration of peripheral blood mtDNA content has also been found to be associated with tumor progression and patient prognosis. For example, lower peripheral blood leukocyte mtDNA copy number was correlated with aggressiveness and fast progression of prostate cancer^14^. Also, high mtDNA copy number in peripheral blood may predict a poor prognosis for hepatocellular carcinoma^15^, glioma^16^, colorectal cancer^17^, and head and neck cancer^18^. Taken together, these results suggested that the alteration of mtDNA content might play complicated roles in tumorigenesis and progression in a cancer-specific manner.

As above, the involvement of mtDNA content changes in carcinogenesis remains controversial and ambiguous. Meanwhile, a single study might have been underpowered to detect the overall effects. Thus in this study we carried out a meta-analysis to summarize the existing evidence and to make a more precise evaluation about the relationship between mtDNA copy number and cancer risk among different populations.

## Methods

### Literature search

Relevant studies that estimated the association between mtDNA copy number and risk of cancers were identified from the databases of PubMed, EMBASE, and China National Knowledge Infrastructure (CNKI) up to March 1, 2016 by two investigators independently. The following key words were used for searching: “mitochondrial DNA”, “risk” and “copy number”. Other alternative spellings of these key words such as “content” and “susceptibility” were also used; only those publications in English were retained.

### Inclusion and Exclusion criteria

Studies were considered eligible for the current meta-analysis if they met the following criteria: (1) they assessed the relationship between mtDNA copy number (or mtDNA content) and risk (or susceptibility) of diverse cancer types; (2) used a cohort or case–control studies design; (3) had an appropriate description of mtDNA copy number in cases and controls; and (4) reported an odds ratio (OR) with a 95% confidence interval (CI) or other available data for calculating the OR (95% CI).

Exclusion criteria: (1) review articles and editorials; (2) case reports; (3) studies not focused on the mtDNA content and cancer risk; and (4) investigation of the functional role of mtDNA in carcinogenesis.

### Data extraction

The following information from each eligible study was carefully extracted independently by two investigators: name of the first author, year of publication, country of origin, number of cases and controls, numbers of genotyped cases and controls, mitochondrial DNA haplotypes, subject population (categorized as East Asian, Indian, Western and Africa populations), source of controls (hospital-based [HB] and population-based [PB]), exposure to smoking, alcohol consumption, and body mass index (BMI).

### Statistical analysis

The strength of the association between mtDNA content and risk of diverse cancers was measured by calculating the odds ratio (OR) with a 95% confidence interval (95% CI), and two-tailed P < 0.05 was considered as statistically significant. First, the overall association between mtDNA content and cancer risk was assessed by categorizing mtDNA content into high and low haplotypes based on cut-off points at the median, the 1^st^ tertile and the 2^nd^ quintile. Specifically, for those studies that categorized the mtDNA content into dichotomies or quartiles, the cut-off point was set at the median value in controls. For those studies categorizing mtDNA into tertiles, the 1^st^ tertile was treated as the low mtDNA content group, while the 2^nd^ and 3^rd^ tertiles were seen as the high mtDNA content group. For those studies that categorized mtDNA into quintiles, the 1^st^ and 2^nd^ quintiles were treated as the low content group, while the rest were designated as the high content group. The detailed information for the categorization is presented in supplementary Table S1. Nested case-control studies and case-control studies were then combined to calculate the odds ratio in total and separately.

Further association analysis was performed by recruiting studies of quartiles to test if a dose-dependent relationship existed, under which condition the 1^st^ quartile was designated as the reference. Then we compared the odds ratio for the other quartiles with the reference to test whether the higher mtDNA contents correlated with cancer risk more obviously.

Heterogeneity among studies was further examined with the *I*^*2*^ statistic, which was interpreted as the proportion of total variation contributed by between-study variation. The Mantel–Haenszel method was used to calculate the OR for the pooled data in a fixed effects model if there was no evidence for significant heterogeneity. Otherwise a random effects model was selected to combine the data. Several subgroup meta-analyses were performed in an attempt to assess the association between the mtDNA content and cancer risk based on the population, age, gender, smoking status, alcohol drinking and BMI.

A sensitivity analysis was also performed to examine the reliability of our study by excluding individual studies orderly. Cumulative analysis was performed to explore the tendency of the changes in risk association by publication year. Evidence of publication bias was determined by visual inspection of the funnel plot and Egger’s test. All statistical analyses were performed with STATA software (version 10.0; StataCorp, College Station, TX, USA). All P values are two-sided.

## Results

### Characteristics of eligible studies

Finally, a total of 36 articles involving 11,847 cases and 15,438 controls were used in the current meta-analysis. By the sample sources according to original records, we classified these 36 articles into 39 independent study subgroups, among which there were twenty-five studies from Western populations, eleven from East Asia, two from India and one from Africa (Table 1). There were five studies with hospital-based controls and the others included population-based controls.

Among these 36 studies, five each focused on breast cancer, four each on renal clear cell carcinoma, three each on lung cancer, colorectal cancer, lymphoma and hepatic carcinoma /cirrhosis, two each on glioma, melanoma, oral and gastric cancer, and one each on nasopharyngeal cancer, endometrial cancer, prostate cancer, esophageal carcinoma, pancreatic carcinoma, soft tissue sarcoma and bladder cancer.

Most studies used ND1 and D-loop which were located on mtDNA as target regions to determine mtDNA copy number. Hemoglobin subunit beta (HBB), human globulin (HGB), 18 s RNA and glyceraldehyde-3-phosphate dehydrogenase (GAPDH) were the most widely used reference genes, and they are widely accepted single copy nuclear genes. All the information about the measurements applied in the studies can be obtained in supplementary Table S1.

### Association between mtDNA content and cancer risk in overall and stratified analyses

**Figure 1:**
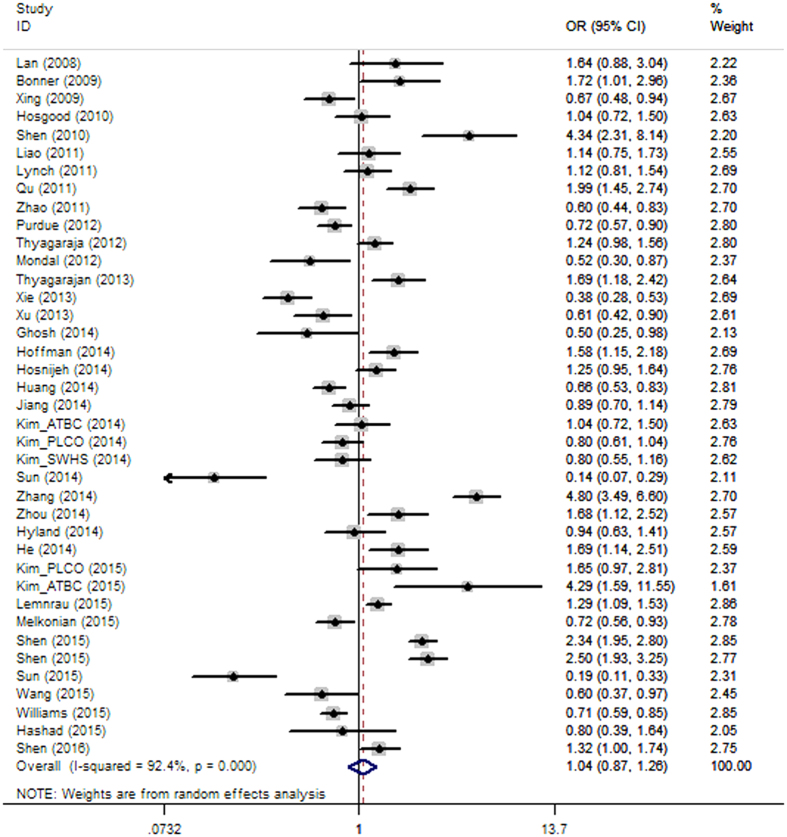
Forest plot of the ORs for the overall cancer risk in dichotomizing analysis. The squares and horizontal lines correspond to the study-specific OR and 95% CI.

When all studies were pooled for the overall meta-analysis by dichotomizing mtDNA copy number into high and low content groups, there was no significant association between mtDNA content and overall cancer risk (OR = 1.044, 95% CI 0.866–1.260, P = 0.651), and there was high heterogeneity (*I*^*2*^ = 92.4%), which may be explained by different cancer types and populations (Fig. 1).

Results of the subgroup analyses are presented in Table 2. Stratification analysis by cancer types identified increased cancer risk associated with high mtDNA content in the subgroups of breast cancer (OR = 1.721, 95% CI = 1.130–2.622, p = 0.011) and lymphoma (OR = 1.645, 95% CI = 1.117–2.421, p = 0.012 respectively), with high and moderate heterogeneity, respectively (*I*^*2*^ = 92.6% and 51.6%). Marginal increased risk associated with high mtDNA content was found in melanoma and glioma, with an odds ratio of 1.555 (95% CI = 0.598–4.045, p = 0.365, *I*^*2*^ = 93.6%) and 2.510 (95% CI = 0.709–8.887, p = 0.154, *I*^*2*^ = 97.2%), respectively. In contrast, mtDNA content was negatively significantly correlated with hepatic malignancy (OR = 0.622, 95% CI = 0.486–0.798, p = 0.000). We further categorized the studies by organ systems, such as digestive, respiratory, gynecological and urinary carcinoma. However, no significant association was identified in any group.

Further subgroup analyses were performed by stratifying studies according to population, age, and gender, as well as life styles such as smoking status, alcohol consumption and body mass index (Table 2). The results revealed that high mtDNA content may predict increased carcinogenesis risk in people with drinking addiction (OR = 1.261, 95% CI = 1.076–1.479, p = 0.004). A total of five studies that provided drinking-related data were included for stratified analysis, and it is notable that four of these studies came from digestive cancers, indicating that high mtDNA content accompanied by drinking addiction may contribute to elevated digestive cancer risk.

According to the study design, we classified the studies into nested case-control and case-control studies (supplementary Table S1), and we performed association analysis based on the study design as well (Table 3). The results from the nested case-control and case-control study did not differ significantly from the results above, which demonstrated the stability and reliability of our analysis. It was surprising to find that mtDNA content was negatively significantly correlated with renal cancer risk in the case-control studies, which was obscured in the stratified analysis in the previous section.

### Cancer risk increased as mtDNA content elevated by quartile analyses

To investigate the possible dose-dependent relationship between mtDNA content and cancer risk, we enrolled the studies that categorized mtDNA copy number into quartiles for further analysis and assigned the 1^st^ quartile as the reference. A total of 20 studies were included in the dose-dependent relationship analyses. No significant difference was found in the risk among carriers of the 2^nd^, 3^rd^ and 4^th^ quartiles in the overall analysis. However, stratified analysis by cancer types found that carriers of higher mtDNA content exhibited gradually increasing risk for breast cancer and melanoma (Table 4), which is consistent with the findings in the dichotomous meta-analysis, further supporting the idea that high mtDNA predisposes increased risk in breast cancer and melanoma. After grouping studies by population, the association of mtDNA content in 4^th^ quartiles with cancer risk was more obvious in western populations than in Asians (OR = 1.122, p = 0.016 versus OR = 0.981, p = 0.896). There were no obvious differences in cancer risk among males and females or among frequent and infrequent smokers.

### Cumulative analysis

**Figure 2:**
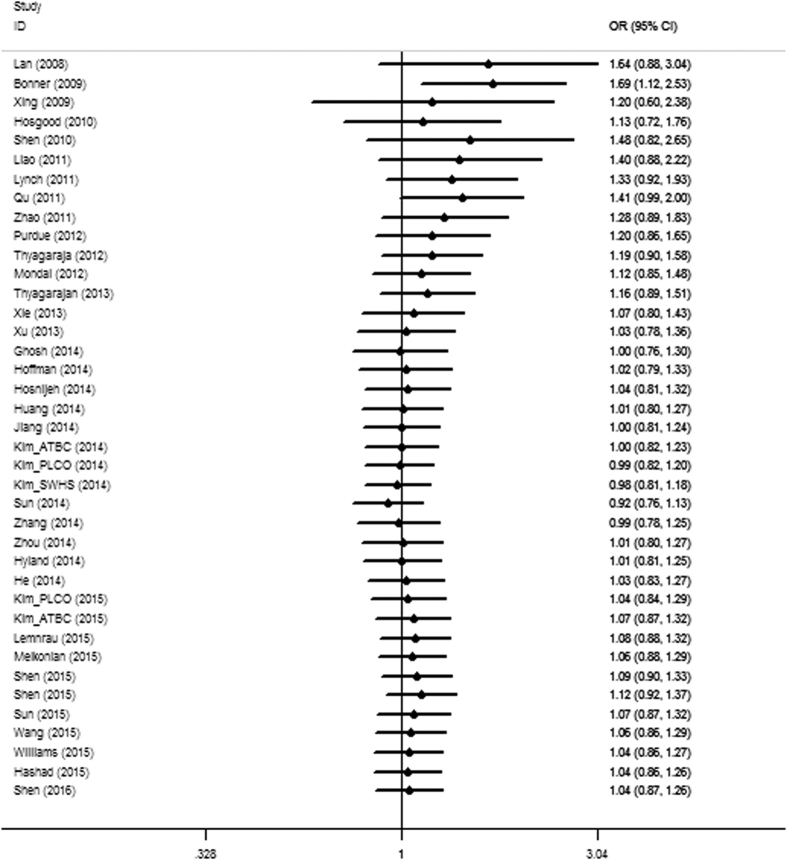
Cumulative analysis of the overall ORs. The results were sorted by publication years.

The findings from the cumulative meta-analysis showed that the positive association was more obvious in early studies but decreased as the data increased by publication year, and the association strength remained stable in the 95% confidence intervals with increasing sample sizes in recent years (Fig. 2), which further demonstrated the complicated roles of mtDNA content in diverse carcinogenesis.

### Sensitivity analyses

**Figure 3:**
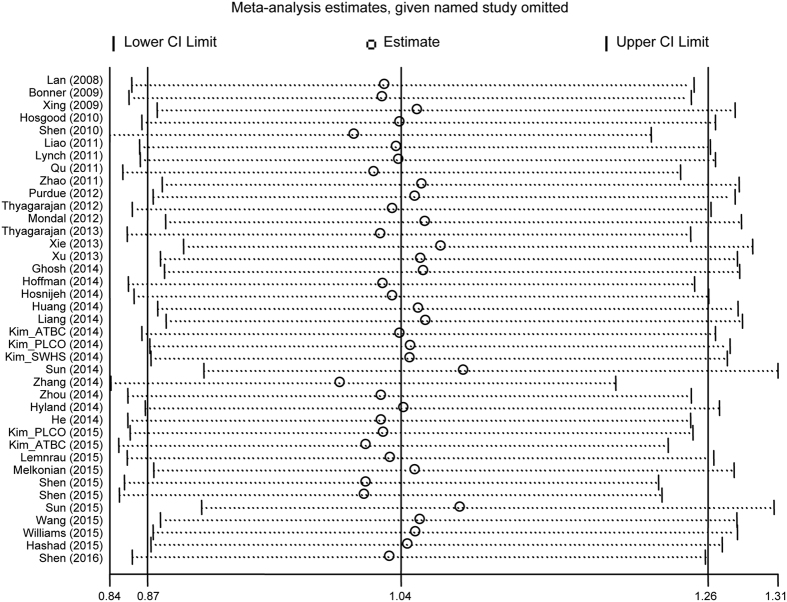
Sensitivity analysis of the summary ORs on the association between mtDNA content and overall cancer risk. The results were calculated by omitting each eligible study. Meta-analysis random-effects estimates were used.

A sensitivity analysis was conducted to assess the influence of each study by sequential omission of each eligible study both in the overall meta-analysis and stratified analysis. The results showed that the significance of the OR was not affected by any single study (Fig. 3), indicating the reliability of our study.

### Evaluation of publication bias

**Figure 4:**
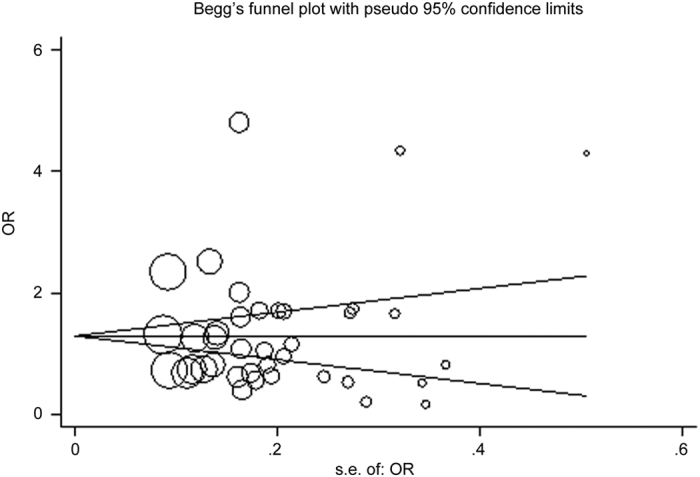
Begg’s funnel plot for publication bias analysis. Each point represents a separate study for the indicated association. Log[or], natural logarithm of the odds ratio. Horizontal line indicates effect size.

Both Begg’s funnel plot and Egger’s test were performed to assess the publication bias. The shape of the funnel plots did not reveal any evidence of obvious asymmetry for any genetic model in the overall meta-analysis (Fig. 4). Next, Egger’s test was used to provide statistical evidence for the funnel plot symmetry, and the results still did not suggest any obvious evidence of publication bias (P = 0.868). Thus, there was no obvious risk of publication bias in this meta-analysis.

## Discussion

Mitochondria are energy factories that produce adenosine triphosphate (ATP) through oxidative phosphorylation. Both increased and decreased mtDNA copy number in blood have been previously reported to be associated with cancer risk in a cancer-specific manner. For example, it has been reported that increased mtDNA copy number contributes to high risk for breast cancer and lymphoma, while decreased mtDNA content was relevant to renal and colorectal cancers. The opposite effects brought out by altered mtDNA content may represent diverse biological roles that mtDNA plays in malignancies.

Biologically, lowered mtDNA copy number can cause deficiency in oxidative phosphorylation and enhanced production of toxic metabolites in aerobic metabolism and glycolysis, resulting in the disruption of cellular functions. Also, it has been demonstrated that mtDNA reduction promotes cancer cells to become more resistant to apoptosis, and leads to epithelial-mesenchymal transition (EMT)^19^,^20^, an important process by which epithelial cells lose their cell polarity and cell-cell adhesion and acquire mesenchymal, fibroblast-like characteristics, accompanied by increased cell migratory and invasive properties, thus endowing the incipient cancer cell with invasive and metastatic properties^21^. On the other hand, high mtDNA content can be an indicative marker of oxidative stress, which is associated with environmental exposure to pollutants, tobacco, smoke, drugs, xenobiotics, or radiation. High mtDNA content is also an important marker of an impaired aerobic mechanism that is thought to be involved in the molecular mechanisms of carcinogenesis^22^. These observations together suggest a complicated role for the changes in mtDNA copy number on the modulation of cancer risk. In the present study, we performed this meta-analysis to achieve a more conclusive result.

Our analysis included 36 studies that focused on diverse cancer types from different origins around the world^23^,^24^,^25^,^26^,^27^,^28^,^29^,^30^,^31^,^32^,^33^,^34^,^35^,^36^,^37^,^38^,^39^,^40^,^41^,^42^,^43^,^44^,^45^,^46^,^47^,^48^,^49^,^50^,^51^,^52^,^53^,^54^,^55^,^56^,^57^,^58^. To reduce the uncertainty induced by confusing factors in the meta-analysis, we evaluated the association between mtDNA content with cancer risk in an overall analysis, as well as stratified analyses by cancer types, genders, ages, ethnicity and life styles.

Finally, we did not find an obvious association between mtDNA content and cancer risk in the overall analysis, which should be the result of the heterogeneity among different cancer types. Thus subgroup analyses were performed to further detect the association strength. Stratified analysis by cancer types provided evidence on the relationship between high mtDNA content and increased breast cancer and lymphoma risk. Dose-dependent effects were also found for melanoma risk. The biological contribution of increased mtDNA content in the peripheral blood to carcinogenesis remains obscured. Previous studies found a correlation between mtDNA content and markers of oxidative stress, such as thiobarbituric acid reactive substances and 8-hydroxyguanosine^59^. Moreover, lower levels of antioxidants were also detected in blood with an increased mtDNA copy number. Due to lack of sufficient protective mechanisms, mtDNA mutations may occur during the process of oxidative stress, and certain mtDNA mutations may lead to the generation of increased superoxide and nitric oxide, resulting in aberrant mitochondrial biogenesis^60^, which has been associated with deficient or defective apoptosis, and confers a replicative advantage to the cells^59^. Therefore it is plausible that high mtDNA content in peripheral blood leukocytes may be indicative of increased oxidative stress, impaired aerobic metabolism, and ROS-mediated DNA damage. mtDNA copy number may increase to compensate for mtDNA damage and mitochondrial dysfunction^3^. Thus it is more likely that the increased mtDNA content is a significant marker of carcinogenesis associated oxidative stress, but not a cause of cancer development.

Stratified analysis also found a significant negative association between mtDNA content and susceptibility of hepatic carcinoma, suggesting the complexity of the involvement of mtDNA changes in carcinogenesis in different populations, which should not be explained by any single mechanism. It has been demonstrated that mtDNA decrease alters mitochondrial gene expression, resulting in deficiency in oxidative phosphorylation, and causes a disturbance of cellular functions. Warburg proposed that damage to the respiratory chain was a critical event in carcinogenesis^61^, which demonstrates that cancer cells enhanced the generation of adenosine triphosphate by glycolysis^62^, resulting in a stronger tolerance to hypoxia and reducing the dependence of mitochondrial oxidative phosphorylation, thus conferring tumor cells the advantages of growth. Enhanced glycolysis also leads to excessive production of lactate and prosurvival proteins, which may initiate and promote cancer development. MtDNA reduction was also found to increase cancer cells’ resistance to apoptosis and lead to epithelial-mesenchymal transition, which are both common in tumor formation and metastatic progression. Some previous studies have partially explained the mechanism by which mtDNA reduction acts on apoptosis resistance and epithelial-mesenchymal transition. Biswas *et al*. reported that decreased mtDNA content could activate NFkappaB/Rel factors^63^. Activation of nuclear factor-kappa B signaling plays a critical role in apoptosis resistance and is clearly linked to various cancer malignant transformation^64^. In addition, activation of the AKT pathway by an mtDNA deficiency could also inhibit cell apoptosis^65^,^66^. As for the contribution to EMT, mtDNA loss may activate PI3K/Akt2, and Raf/MAPK pathways, which may finally lead to EMT and cancer metastasis^67^,^68^.

Stratified analysis by life styles, such as smoking, drinking and BMI status, suggested alcohol users with high mtDNA content may be more susceptible to cancer development, indicating an intrinsic relationship between mtDNA and alcohol degradation. Surprisingly, we did not find any association between altered mtDNA and smoking-related malignancies, although it has been widely accepted that smoking is an important stimulator of diverse cancers. Previous evidence has shown that the metabolites of cigarettes should be oxidative phosphorylated by mitochondria, so we proposed that abnormal mtDNA content may predict altered cancer risk in the smoking population. However, we did not obtain the suspected results, indicating that tumor-genesis of smoking related cancers involves much more cellular abnormality than mtDNA changes.

In summary, our study provides evidence that increased mtDNA content is statistically significantly associated with risk of lymphoma, breast cancer and melanoma, but is negatively associated with hepatic carcinoma. The mechanism for the tumor specific associations between mtDNA content and cancer risk remain to be illustrated, although they are likely to be regulated by a wide range of genetic, molecular, and cellular determinants. For instance, elevated mtDNA content has been significantly associated with altered oxidative stress, aging, immune response activation, and response to environmental exposure. Future studies are warranted to evaluate oxidative stress related factors that influence mtDNA copy number and to provide novel insights into the biological mechanisms of mtDNA copy number variation on the development of various cancers, especially on the opposite effects of increased and decreased mtDNA content in carcinogenesis. Furthermore, many more subjects are needed to confirm the association identified by our meta-analysis in different populations.

## Additional Information

**How to cite this article**: Hu, L. *et al*. Altered mitochondrial DNA copy number contributes to human cancer risk: evidence from an updated meta-analysis. *Sci. Rep.* **6**, 35859; doi: 10.1038/srep35859 (2016).

## Supplementary Material

Supplementary Information

## Figures and Tables

**Table 1:** Study and host characteristics included in this meta-analysis.

Author	Year	Cancer type	Sample source	Country	Ethnicity	Sample size Ca/Co	Gender		Age, year old Ca/Co	BMI, kg/m^2^ Ca/Co Ca/Co	Smoking status		Drinking status	
							Male Ca/Co	Female			Ever Ca/Co	Never Ca/Co	Ever Ca/Co	Never Ca/Co
Lan	2008	lymphoma	Population	Finland	Caucasian	104/104	Un	Un	58/57	25.5/25.4	Un	Un	Un	Un
Shen	2010	Breast cancer	Population	USA	Caucasian	103/103	Un	103/103	58/56	27.8/26.4	52/51	Un	75/78	Un
Xing	2009	Renal cell carcinoma	Population	USA	Caucasian	260/281	172/175	88/106	Un	Un	134/126	125/155	Un	Un
Bonner	2009	Lung cancer	Population	China	Asian	113/107	73/68	40/39	54.9/54.5	Un	Un	Un	Un	Un
Hosgood	2010	Lung cancer	Population	Finland	Caucasian	227/227	227/227	0/0	58.7/58.4	25.6/26.3	227/227	0/0	Un	Un
Liao	2011	Gastric cancer	Population	China	Asian	162/299	0/0	162/299	61.0/61.0	Un	Un	Un	Un	Un
Lynch	2011	pancreatic	Population	Finland	Caucasian	203/656	203/656	0/0	58.0/58.0	26.2/25.7	203/656	0/0	Un	Un
Qu	2011	Colorectal	Population	China	Asian	320/320	169/151	169/151	58.4/58.2	23.8/23.6	126/105	194/215	42/35	278/285
Zhao	2011	Hepatocellular carcinoma	Population	China	Asian	274/384	236/218	38/40	50.1/48.7	Un	154/132	120/126	98/42	176/216
Purdue	2012	Renal cell carcinoma	Population	USA	Caucasian	445/379	256/247	189/132	Un	Un	293/231	152/148	Un	Un
			Population		African	158/224	109/110	49/114	Un	Un	112/154	46/70	Un	Un
Thyagarajan	2012	Colorectal	Population	Singapore Chinese	Asian	422/874	250/380	172/494	66.1/57.6	23.0/22.8	181/228	241/646	Un	Un
Mondal	2012	Oral	Hospital	India	Asian	124/140	98/26	101/39	58/56	Un	89/75	35/65	Un	Un
Thyagarajan	2013	Breast	Population	Singapore Chinese	Asian	183/529	0/0	183/529	61.1/61.1	Un	Un	Un	Un	Un
Xie	2013	Soft tissue sarcoma	Population	USA	Mixed	325/330	174/180	151/150	58.24/58.50	Un	122/117	174/212	Un	Un
Xu	2013	Esophageal adenocarcinoma	Population	USA	Caucasian	218/218	173/173	45/45	62.05/60.94	Un	150/122	68/96	Un	Un
Ghosh	2014	Nasopharyngeal carcinoma	Hospital	India	Asian	64/100	49/79	15/21	Un	Un	45/26	19/74	Un	Un
Hoffman	2014	Renal cell carcinoma	Population	USA	Caucasian	230/468	164/331	66/137	Un	Un	212/256	92/138	Un	Un
Hosnijeh	2014	lymphoma	Population	European	Caucasian	469/469	231/231	238/238	56.6/56.6	26.9/26.6	272/264	197/205	Un	Un
Huang	2014	Colorectal cancer	Population	China	Asian	444/1423	0/0	444/1423	58.6/55.2	24.6/24.4	11/46	433/1377	14/39	430/1384
Jiang	2014	Breast cancer	Population	China	Asian	506/520	0/0	506/520	Un	Un	Un	Un	Un	Un
Kim ATBC	2014	Lung cancer	Population	Finland	Caucasian	227/227	227/227		56.67/58.41	25.59/26.35	227/227	Un	Un	Un
Kim PLCO			Population	USA	Mixed	426/436	259/267	167/169	64.07/63.67	26.84/27.43	380/238	Un	Un	Un
Kim SWHS			Population	China	Asian	221/222	0/0	221/222	59.2/59.2	24.58/25.01	17/11	Un	Un	Un
Sun	2014	gastric	Population	USA	Caucasian	132/125	74/65	58/60	58.2/55.5	Un	55/82	77/43	Un	Un
Zhang	2014	glioma	Population	China	Asian	414/414	241/241	173/173	Un	Un	96/85	318/329	Un	Un
Zhou	2014	Prostate cancer	Population	China	Asian	193/194	193/194	0/0	70.3/70.1	24.1/23.2	100/98	93/96	Un	Un
Hyland	2014	Melanoma	Population	USA	Mixed	136/302	130/63	172/73	Un	Un	Un	Un	Un	Un
Kim ATBC	2015	lymphoma	Population	Finland	Caucasian	142/142	142/142	Un	Un	Un	142/142	0/0	Un	Un
Kim PLCO	2015	lymphoma	Population	USA	Mixed	292/301	178/185	114/116	Un	Un	135/155	157/146	Un	Un
Lemnrau	2015	Breast Cancer	Population	UK	Caucasian	1053/1053	Un	Un	Un	Un	428/410	679/687	981/936	125/160
Melkonian	2015	Renal cell carcinoma	Population	USA	Caucasian	608/629	400/418	208/211	58.46/58.57	Un	297/287	311/342	Un	Un
Shen	2015	breast	Population	USA	Caucasian	1000/1000	Un	1000/1000	58/56	27.8/26.4	315/288	685/712	720/750	280/250
Shen	2015	melanoma	Population	USA	Mixed	500/500	238/262	238/262	Un	Un	Un	Un	Un	Un
He	2014	Oralpremalignant lesion	Population	USA	Mixed	143/357	87/215	56/142	57.3/58.5	Un	83/175	60/182	85/108	53/249
Sun	2015	Endometrial	Hospital	USA	Caucasian	139/139	Un	Un	62.24/61.76	Un	31/48	108/91	Un	Un
Wang	2015	Liver Cirrhosis	Hospital	USA	Caucasian	136/136	112/105	24/31	54.17/54.03	Un	55/54	81/82	68/60	68/76
Williams	2015	Bladder cancer	Hospital	USA	Caucasian	926/926	738/738	188/188	Un	Un	678/521	248/405	Un	Un
Hashad	2015	Liver Carcinoma/Cirrhosis	Population	Egypt	African	90/45	52/30	38/15	50.88/50	Un	Un	Un	Un	Un
Shen	2016	Glioma	Population	USA	Caucasian	395/425	242/221	149/204	49/52	28.2/27.6	175/216	198/204	Un	Un

Un: Unknown data.

**Table 2:** Overall and subgroup analyses of mtDNA copy number with cancer risk by dichotomizing mtDNA copy number into high and low groups.

Subgroups	Research counts (n)	Test of association		95% CI	P	Heterogeneity		*I*^2^	*Tau-squared*
		Model	OR			*χ*	*p*		
**Overall**	39	Random	1.044	0.866-1.260	0.651	499.08	0.000	92.4%	0.3139
Cancer types									
Lymphoma	4	Random	1.645	1.117-2.421	0.012^a^	6.20	0.102	51.6%	0.0769
Breast cancer	5	Random	1.721	1.130-2.622	0.011^a^	54.16	0.000	92.6%	0.2015
Lung cancer	5	Random	0.982	0.788-1.223	0.871	7.84	0.097	49.0%	0.2015
Colorectal cancer	3	Random	1.168	0.638-2.139	0.641	34.38	0.000	94.2%	0.2681
Renal carcinoma	4	Random	0.856	0.598-1.226	0.397	19.71	0.000	84.8%	0.1126
Melanoma	2	Random	1.555	0.598-4.045	0.365	15.73	0.000	93.6%	0.4456
Glioma	2	Random	2.510	0.709-8.887	0.154	35.96	0.000	97.2%	0.8091
Gastric	2	Random	0.412	0.054-3.134	0.392	25.76	0.000	96.1%	2.0587
Hepatic	3	Fixed	0.622	0.486-0.798	0.000^a^	0.53	0.767	0.0%	
Oral	2	Random	0.946	0.295-3.029	0.925	12.43	0.000	92.0%	0.6489
Organ systems									
Digestive system	12	Random	0.861	0.632-1.173	0.344	95.74	0.000	88.5%	0.2521
Urogenital system	6	Random	0.876	0.635-1.209	0.420	43.03	0.000	88.4%	0.1398
Respiratory system	6	Random	0.928	0.730-1.179	0.539	11.74	0.039	57.4%	0.0488
Gynecologica system	6	Random	1.253	0.744-2.109	0.396	106.55	0.000	95.3%	0.3847
Population									
Western	25	Random	0.951	0.719-1.259	0.728	456.55	0.000	94.7%	0.4628
East Asian	11	Random	1.296	0.901-1.866	0.162	147.15	0.000	93.2%	0.3481
India	2	Fixed	0.508	0.335-0.771	0.001	0.01	0.938	0.0%	
Africa	1		0.799						
Age^b^									
Elder	10	Random	1.104	0.655-1.860	0.710	111.22	0.000	91.9%	0.6352
Younger	10	Random	1.079	0.670-1.738	0.754	95.11	0.000	90.5%	0.5213
Gender									
Male	16	Random	1.036	0.755-1.420	0.828	155.98	0.000	90.4%	0.3578
Female	22	Random	1.109	0.866-1.420	0.414	191.26	0.000	89.0%	0.2788
Life styles									
Smoking status									
Ever/Frequent	21	Random	0.984	0.765-1.267	0.902	106.17	0.000	81.2%	0.2465
Never/Occasionally	18	Random	1.047	0.736-1.491	0.798	165.82	0.000	89.7%	0.4972
Drinking Status									
Ever/Frequent	5	Fixed	1.261	1.076-1.479	0.004^a^	4.53	0.340	11.6%	
Never/Occasionally	5	Random	1.179	0.971-1.432	0.096	42.69	0.000	90.6%	0.5403
BMI									
High^c^	4	Random	1.103	0.413-2.942	0.845	36.84	0.000	91.9%	0.9038
Low^c^	4	Random	1.077	0.550-2.109	0.828	15.12	0.002	80.2%	0.3609

^a^p < 0.05;

^b^Age was classified as elder and younger according to publication records; ^c^Relative value which means relatively high and relatively low BMI.

**Table 3:** Comparison of cancer risk association between nested case-control and case-control studies.

Subgroups	Research counts (n)	Test of association		95% CI	P	Heterogeneity		*I*^2^	*Tau-squared*
		Model	OR			*χ*	*p*		
Overall									
*Nested case-control*	16	Random	1.133	0.957-1.342	0.651	62.08	0.000	75.8%	0.0816
Case-control	23	Random	0.958	0.708-1.297	0.783	436.96	0.000	95.0%	0.5011
Cancer types									
*Lymphoma*^a^	4	Random	1.645	1.117-2.421	0.012^c^	6.20	0.102	51.6%	0.0769
*Breast cancer*	2	Fixed	1.360	1.165-1.587	0.000^c^	1.77	0.183	43.6%	
*Breast cancer*^b^	3	Random	2.001	0.897-4.468	0.090	46.98	0.000	95.7%	0.4645
*Lung cancer*	4	Random	0.888	0.752-1.049	0.163	2.29	0.514	0.0%	0.2015
*Lung cancer*	1		0.669						
*Colorectal cancer*	2	Random	0.891	0.760-1.045	0.156	14.68	0.000	93.2%	
*Colorectal cancer*	1		1.991						
*Renal carcinoma*	1		1.584						
*Renal carcinoma*	3	Fixed	0.711	0.611-0.827	0.000^c^	0.15	0.926	0.0%	
*Melanoma*	2	Random	1.555	0.598-4.045	0.365	15.73	0.000	93.6%	0.4456
*Glioma*	2	Random	2.510	0.709-8.887	0.154	35.96	0.000	97.2%	0.8091
*Gastric*	2	Random	0.412	0.054-3.134	0.392	25.76	0.000	96.1%	2.0587
*Hepatic*	1		0.600						
*Hepatic*	2	Fixed	0.630	0.472-0.842	0.002^c^	0.50	0.479	0.0%	
*Oral*	2	Random	0.946	0.295-3.029	0.925	12.43	0.000	92.0%	0.6489
Organ systems									
*Digestive system*	5	Random	0.920	0.673-1.258	0.602	20.22	0.204	80.2%	0.0975
*Digestive system*	7	Random	0.711	0.399-1.266	0.247	75.74	0.000	92.1%	0.5452
*Urogenital system*	1		1.584						
*Urogenital system*	5	Random	0.808	0.637-1.025	0.079	16.11	0.003	75.2%	0.0533
*Respiratory system*	4	Random	0.888	0.752-1.049	0.163	2.29	0.514	0.0%	0.2015
*Respiratory system*	2	Random	0.942	0.279-3.181	0.924	7.97	0.005	87.4%	0.6742
*Gynecologica system*	2	Fixed	1.360	1.165-1.587	0.000^a^	1.77	0.183	43.6%	
*Gynecologica system*	4	Random	1.143	0.443-2.949	0.782	103.65	0.000	97.1%	0.8848

^a^The bold inclined letters indicate the nested case-control study.

^b^The thin inclined letters indicate the case-control study.

^c^p < 0.05.

**Table 4:** Overall and subgroup analyses of mtDNA copy number with cancer risk in quartiles.

Study groups	Studies (n)	4^th^ versus 1^st^ quartile		p	*I*^*2*^	3^rd^ versus 1^st^ quartile		p	*I*^*2*^	2^nd^ versus 1^st^ quartile		p	*I*^*2*^
		OR	95%CI			OR	95%CI			OR	95%CI		
Overall	21	1.025	0.765-1.373	0.870	90.4%	0.923	0.743-1.146	0.466	81.9%	0.925	0.777-1.101	0.382	71.9%
Cancer types													
Breast cancer	2	1.906	0.974-3.729	0.060	92.9%	1.573	0.973-2.542	0.065	85.8%	1.250	0.811-1.927	0.312	81.8%
Lung cancer	4	1.068	0.783-1.456	0.677	40.5%	0.816	0.640-0.040	0.100	0.0%	1.111	0.880-1.401	0.376	0.0%
Renal cancer	2	0.594	0.456- 0.775	0.000	97.4%	0.572	0.437-0.749	0.000	96.2%	0.511	0.387-0.674	0.000	93.0%
Melanoma	2	1.792	1.313-2.445	0.000	87.9%	1.696	1.240-2.320	0.001	49.9%	1.544	1.124-2.119	0.007	10.2%
Organ systems													
Digestive cancers	5	0.997	0.806-1.233	0.978	78.7%	0.673	0.537-0.845	0.001	65.7%	0.622	0.495-0.780	0.000	48.3%
Urogenital cancers	4	0.788	0.663-0.938	0.007	92.0%	0.860	0.724-1.021	0.085	84.2%	0.864	0.726-1.026	0.096	57.8%
Population													
Western	14	1.122	1.022-1.233	0.016	90.8%	0.916	0.831-1.008	0.073	84.1%	0.881	0.799-0.971	0.010	74.7%
East Asian	5	0.981	0.739-1.303	0.896	80.8%	0.962	0.727-1.272	0.784	73.0%	0.962	0.727-1.272	0.784	73.0%
Gender													
Male	6	0.762	0.622-0.934	0.009	90.7%	0.662	0.535-0.819	0.000	63.7%	0.768	0.625-0.944	0.012	48.0%
Female	6	0.720	0.563-0.920	0.009	80.0%	0.750	0.588-0.957	0.020	55.2%	0.733	0.572-0.939	0.014	70.5%
Life styles													
Smoking staus													
Ever/Frequent	5	1.121	1.005-1.250	0.040	43.5%	1.031	0.918-1.158	0.603	57.0%	1.036	0.919-1.167	0.563	0.0%
Never/occasionally	5	1.117	1.001-1.247	0.048	0.0%	1.054	0.939-1.182	0.372	0.0%	1.018	0.906- 1.144	0.761	0.0%
Drinking status													
Ever/Frequent	1	1.149	1.024-1.289	0.018		1.075	0.953-1.212	0.242		0.998	0.875-1.138	0.974	
Never/occasionally	1	1.137	0.790-1.635	0.489		1.160	0.862-1.562	0.328		1.040	0.749-1.444	0.814	
BMI													
High	1	1.398	1.131-1.728	0.002		1.227	0.976-1.543	0.080		1.053	0.796-1.391	0.719	
Low	1	0.950	0.609-1.481	0.820		1.058	0.700-1.599	0.790		1.023	0.665-1.574	0.917	
