# Supplementary material for: Altered mitochondrial DNA copy number contributes to human cancer risk: evidence from an updated meta-analysis
Source: Sci Rep. 2016 Oct 24;6:35859. doi: 10.1038/srep35859 (PMC5075889; doi:10.1038/srep35859)
Supplement: Supplementary Information [file srep35859-s1.doc]

**Altered mitochondrial DNA copy number contributes to human cancer risk: evidences from an updatedmeta-analysis**

Liwen Hu^1^, Xinyue Yao^2^, Yi Shen^1^

Liwen Hu and Xinyue Yao contributed equally to this work.

^1^ Department of Cardiothoracic Surgery, Jinling Hospital, School of Clinical Medicine,Nanjing University, Nanjing, Jiangsu Province, PR China;

^2^ Institute of Laboratory Medicine, Jinling Hospital, School of Clinical Medicine, Nanjing University, Nanjing,Jiangsu Province, PR China;

E-mail:

Liwen Hu: [hoofu@163.com](mailto:hoofu@163.com)

Xinyue yao: 710950783@qq.com

Yi Shen: [prof_yishen@sina.com](mailto:prof_yishen@sina.com)

Corresponding Author: Yi Shen; Department of Cardiothoracic Surgery, Jinling Hospital, Zhongshan East road, Xuanwu district, Nanjing, Jiangsu Province, People’s Republic of China, 210002.

E-mail: prof_yishen@sina.com

Tel/ Fax: +862580861012

Table S1. Supplementary information of included studies

| Study name | study design | categorization | cases number | | controls number | | detection method | target gene | reference gene | Sample types |
| --- | --- | --- | --- | --- | --- | --- | --- | --- | --- | --- |
|  |  |  |  |  |  |  |  |  |  |  |
|  |  |  | high | low | high | low |  |  |  |  |
| Lan 2008 | nested | By tertiles |  |  |  |  | qPCR | ND1 | HBB | Blood |
|  |  | 1st |  | 23 |  | 33 |  |  |  |  |
|  |  | 2nd | 36 |  | 36 |  |  |  |  |  |
|  |  | 3rd | 45 |  | 35 |  |  |  |  |  |
|  |  |  |  |  |  |  |  |  |  |  |
| Shen 2010 | case-control | By median | 84 | 19 | 52 | 51 | qPCR | ND1 | HGB | Blood |
|  |  |  |  |  |  |  |  |  |  |  |
| Xing 2009 | case-control | By median | 102 | 158 | 138 | 143 | qPCR | ND1 | HGB | Blood |
|  |  |  |  |  |  |  |  |  |  |  |
| Bonner 2009 | case-control | By median | 72 | 41 | 54 | 53 | qPCR | ND1 | HGB | Spum |
|  |  |  |  |  |  |  |  |  |  |  |
| Hosgood 2010 | nested | By quartile |  |  |  |  | qPCR | ND1 | HGB | Blood |
|  |  | 1st |  | 52 |  | 57 |  |  |  |  |
|  |  | 2nd |  | 59 |  | 56 |  |  |  |  |
|  |  | 3rd | 43 |  | 57 |  |  |  |  |  |
|  |  | 4th | 73 |  | 57 |  |  |  |  |  |
|  |  |  |  |  |  |  |  |  |  |  |
| Liao 2011 | nested | By quartile |  |  |  |  | qPCR | ND1 | HBB | Blood |
|  |  | 1st | 33 |  | 62 |  |  |  |  |  |
|  |  | 2nd | 38 |  | 62 |  |  |  |  |  |
|  |  | 3rd |  | 32 |  | 62 |  |  |  |  |
|  |  | 4th |  | 31 |  | 63 |  |  |  |  |
|  |  |  |  |  |  |  |  |  |  |  |
| Lynch 2011 | nested | By quintiles |  |  |  |  | qPCR | ND1 | HBB | Blood |
|  |  | 1st |  | 38 |  | 133 |  |  |  |  |
|  |  | 2nd |  | 39 |  | 133 |  |  |  |  |
|  |  | 3rd | 30 |  | 142 |  |  |  |  |  |
|  |  | 4th | 43 |  | 130 |  |  |  |  |  |
|  |  | 5th | 53 |  | 118 |  |  |  |  |  |
|  |  |  |  |  |  |  |  |  |  |  |
| qu 2011 | case-control | By median | 213 | 107 | 160 | 160 | qPCR | ND1 | HGB | Blood |
|  |  |  |  |  |  |  |  |  |  |  |
| Zhao 2011 | case-control | By median | 103 | 171 | 192 | 192 | qPCR | ND1 | HGB | Blood |
|  |  |  |  |  |  |  |  |  |  |  |
| Purdue 2012 | case-control | By quartile |  |  |  |  | qPCR | ND1 | HBB | Blood |
|  |  | 1st | 119 |  | 150 |  |  |  |  |  |
|  |  | 2nd | 133 |  | 150 |  |  |  |  |  |
|  |  | 3rd |  | 140 |  | 150 |  |  |  |  |
|  |  | 4th |  | 211 |  | 151 |  |  |  |  |
|  |  |  |  |  |  |  |  |  |  |  |
| Thyagarajan 2012 | nested | By quartile |  |  |  |  | qPCR | ND1 | 18s | Blood |
|  |  | 1st |  | 130 |  | 219 |  |  |  |  |
|  |  | 2nd |  | 59 |  | 219 |  |  |  |  |
|  |  | 3rd | 74 |  | 219 |  |  |  |  |  |
|  |  | 4th | 159 |  | 217 |  |  |  |  |  |
|  |  |  |  |  |  |  |  |  |  |  |
| Mondal 2012 | case-control | By quartile |  |  |  |  | qPCR | D-loop | GAPDH | Blood |
|  |  | 1st |  | 58 |  | 43 |  |  |  |  |
|  |  | 2nd |  | 35 |  | 42 |  |  |  |  |
|  |  | 3rd | 19 |  | 29 |  |  |  |  |  |
|  |  | 4th | 12 |  | 26 |  |  |  |  |  |
|  |  |  |  |  |  |  |  |  |  |  |
| Thyagarajan 2013 | nested | By quintiles |  |  |  |  | qPCR | ND1 | 18s | Blood |
|  |  | 1st |  | 29 |  | 117 |  |  |  |  |
|  |  | 2nd |  | 27 |  | 109 |  |  |  |  |
|  |  | 3rd | 38 |  | 95 |  |  |  |  |  |
|  |  | 4th | 46 |  | 102 |  |  |  |  |  |
|  |  | 5th | 43 |  | 106 |  |  |  |  |  |
|  |  |  |  |  |  |  |  |  |  |  |
| Xie 2013 | case-control | By median | 90 | 235 | 165 | 165 | qPCR | ND1 | HGB | Blood |
|  |  | By quartile |  |  |  |  |  |  |  |  |
|  |  | 1st | 23 |  | 82 |  |  |  |  |  |
|  |  | 2nd | 67 |  | 83 |  |  |  |  |  |
|  |  | 3rd |  | 73 |  | 82 |  |  |  |  |
|  |  | 4th |  | 162 |  | 83 |  |  |  |  |
|  |  |  |  |  |  |  |  |  |  |  |
| Xu 2013 | case-control | By median | 83 | 135 | 109 | 109 | qPCR | ND1 | HGB | Blood |
|  |  | by quartile |  |  |  |  |  |  |  |  |
|  |  | 1st | 43 |  | 54 |  |  |  |  |  |
|  |  | 2nd | 40 |  | 55 |  |  |  |  |  |
|  |  | 3rd |  | 52 |  | 54 |  |  |  |  |
|  |  | 4th |  | 83 |  | 55 |  |  |  |  |
|  |  |  |  |  |  |  |  |  |  |  |
| Ghosh 2014 | case-control | By quartile |  |  |  |  | qPCR | D-loop | GAPDH | Blood |
|  |  | 1st |  | 31 |  | 29 |  |  |  |  |
|  |  | 2nd |  | 15 |  | 27 |  |  |  |  |
|  |  | 3rd | 11 |  | 25 |  |  |  |  |  |
|  |  | 4th | 7 |  | 19 |  |  |  |  |  |
|  |  |  |  |  |  |  |  |  |  |  |
| Hofmann 2014 | nested | By quartile |  |  |  |  | qPCR | ND1 | HGB | Blood |
|  |  | 1st |  | 42 |  | 116 |  |  |  |  |
|  |  | 2nd |  | 47 |  | 118 |  |  |  |  |
|  |  | 3rd | 64 |  | 117 |  |  |  |  |  |
|  |  | 4th | 77 |  | 117 |  |  |  |  |  |
|  |  |  |  |  |  |  |  |  |  |  |
| Hosnijeh 2014 | nested | By quartile |  |  |  |  | qPCR | ND1 | HGB | Blood |
|  |  | 1st |  | 112 |  | 107 |  |  |  |  |
|  |  | 2nd |  | 77 |  | 105 |  |  |  |  |
|  |  | 3rd | 85 |  | 104 |  |  |  |  |  |
|  |  | 4th | 144 |  | 102 |  |  |  |  |  |
|  |  |  |  |  |  |  |  |  |  |  |
| Huang 2014 | nested | By tertiles |  |  |  |  |  |  |  |  |
|  |  | 1st |  | 191 |  | 475 | qPCR | ND1 | BRCA1 | Blood |
|  |  | 2nd | 149 |  | 473 |  |  |  |  |  |
|  |  | 3rd | 104 |  | 475 |  |  |  |  |  |
|  |  |  |  |  |  |  |  |  |  |  |
| Jiang 2014 | case-control | By median | 247 | 259 | 269 | 251 | qPCR | NC_012920 | β2M | Blood |
|  |  |  |  |  |  |  |  |  |  |  |
| Kim 2014 ATBC | nested | By quartile |  |  |  |  | qPCR | ND1 | HGB | Blood |
|  |  | 1st |  | 52 |  | 57 |  |  |  |  |
|  |  | 2nd |  | 59 |  | 56 |  |  |  |  |
|  |  | 3rd | 43 |  | 57 |  |  |  |  |  |
|  |  | 4th | 73 |  | 57 |  |  |  |  |  |
|  |  |  |  |  |  |  |  |  |  |  |
| Kim 2014 PLCO |  | By quartile |  |  |  |  |  |  |  |  |
|  |  | 1st |  | 117 |  | 107 |  |  |  |  |
|  |  | 2nd |  | 119 |  | 110 |  |  |  |  |
|  |  | 3rd | 96 |  | 108 |  |  |  |  |  |
|  |  | 4th | 94 |  | 111 |  |  |  |  |  |
|  |  |  |  |  |  |  |  |  |  |  |
| Kim 2014 SWHS |  | By quartile |  |  |  |  |  |  |  |  |
|  |  | 1st |  | 54 |  | 56 |  |  |  |  |
|  |  | 2nd |  | 72 |  | 58 |  |  |  |  |
|  |  | 3rd | 44 |  | 53 |  |  |  |  |  |
|  |  | 4th | 51 |  | 55 |  |  |  |  |  |
|  |  |  |  |  |  |  |  |  |  |  |
| Sun 2014 | case-control | By median | 13 | 90 | 62 | 62 | qPCR | ND1 | HGB | Blood |
|  |  |  |  |  |  |  |  |  |  |  |
| Zhang 2014 | case-control | By median | 342 | 72 | 206 | 208 | qPCR | ND1 | HGB | Blood |
|  |  |  |  |  |  |  |  |  |  |  |
| Zhou 2014 | case-control | By median | 121 | 72 | 97 | 97 | qPCR | ND1 | HGB | Blood |
|  |  | By quartile |  |  |  |  |  |  |  |  |
|  |  | 1st |  | 26 |  | 48 |  |  |  |  |
|  |  | 2nd |  | 42 |  | 49 |  |  |  |  |
|  |  | 3rd | 57 |  | 49 |  |  |  |  |  |
|  |  | 4th | 68 |  | 48 |  |  |  |  |  |
|  |  |  |  |  |  |  |  |  |  |  |
| Hyland 2014 | case-control | By quartile |  |  |  |  | qPCR | ND1 | HBB | Blood |
|  |  | 1st |  | 33 |  | 78 |  |  |  |  |
|  |  | 2nd |  | 37 |  | 73 |  |  |  |  |
|  |  | 3rd | 39 |  | 76 |  |  |  |  |  |
|  |  | 4th | 27 |  | 75 |  |  |  |  |  |
|  |  |  |  |  |  |  |  |  |  |  |
| Kim 2015 PLCO | nested | By tertile |  |  |  |  | qPCR | ND1 | HBB | Blood |
|  |  | 1st |  | 22 |  | 100 |  |  |  |  |
|  |  | 2nd | 38 |  | 98 |  |  |  |  |  |
|  |  | 3rd | 35 |  | 103 |  |  |  |  |  |
|  |  |  |  |  |  |  |  |  |  |  |
| Kim 2015 ATBC |  | By tertile |  |  |  |  |  |  |  |  |
|  |  | 1st |  | 5 |  | 48 |  |  |  |  |
|  |  | 2nd | 14 |  | 45 |  |  |  |  |  |
|  |  | 3rd | 28 |  | 49 |  |  |  |  |  |
|  |  |  |  |  |  |  |  |  |  |  |
| Lemnrau 2015 | nested | By quartile |  |  |  |  | qPCR | ND1 | ALB | Blood |
|  |  | 1st |  | 230 |  | 264 |  |  |  |  |
|  |  | 2nd |  | 234 |  | 267 |  |  |  |  |
|  |  | 3rd | 283 |  | 263 |  |  |  |  |  |
|  |  | 4th | 306 |  | 259 |  |  |  |  |  |
|  |  |  |  |  |  |  |  |  |  |  |
| Melkonian 2015 | case-control | By median | 192 | 243 | 296 | 271 | qPCR | ND1 | HGB | Blood |
|  |  |  |  |  |  |  |  |  |  |  |
| Shen 2015 | case-control | By median | 703 | 317 | 487 | 513 | qPCR | ND1 | HGB | Blood |
|  |  | By quartile |  |  |  |  |  |  |  |  |
|  |  | 1st |  | 142 |  | 256 |  |  |  |  |
|  |  | 2nd |  | 223 |  | 257 |  |  |  |  |
|  |  | 3rd | 274 |  | 245 |  |  |  |  |  |
|  |  | 4th | 361 |  | 242 |  |  |  |  |  |
|  |  |  |  |  |  |  |  |  |  |  |
| Shen 2015 | case-control | By median | 358 | 142 | 251 | 249 | qPCR | ND1 | HGB | Blood |
|  |  | By quartile |  |  |  |  |  |  |  |  |
|  |  | 1st |  | 71 |  | 126 |  |  |  |  |
|  |  | 2nd |  | 122 |  | 125 |  |  |  |  |
|  |  | 3rd | 137 |  | 123 |  |  |  |  |  |
|  |  | 4th | 170 |  | 126 |  |  |  |  |  |
|  |  |  |  |  |  |  |  |  |  |  |
| He 2014 | case-control | By median | 87 | 56 | 171 | 186 | qPCR | ND1 | HGB | Blood |
|  |  |  |  |  |  |  |  |  |  |  |
| Sun 2015 | case-control | By median | 22 | 116 | 70 | 69 | qPCR | ND1 | HGB | Blood |
|  |  |  |  |  |  |  |  |  |  |  |
| Wang 2015 | nested | By median | 51 | 85 | 68 | 68 | qPCR | ND1 | 36B4 | Blood |
|  |  |  |  |  |  |  |  |  |  |  |
|  |  | By quartile |  |  |  |  |  |  |  |  |
|  |  | 1st |  | 25 |  | 34 |  |  |  |  |
|  |  | 2nd |  | 26 |  | 34 |  |  |  |  |
|  |  | 3rd | 35 |  | 34 |  |  |  |  |  |
|  |  | 4th | 50 |  | 34 |  |  |  |  |  |
|  |  |  |  |  |  |  |  |  |  |  |
| Williams 2015 | case-control | By median | 385 | 541 | 463 | 463 | qPCR | ND1 | HGB | Blood |
|  |  | By quartile |  |  |  |  |  |  |  |  |
|  |  | 1st | 167 |  | 231 |  |  |  |  |  |
|  |  | 2nd |  | 218 |  | 232 |  |  |  |  |
|  |  | 3rd |  | 252 |  | 232 |  |  |  |  |
|  |  | 4th |  | 289 |  | 231 |  |  |  |  |
|  |  |  |  |  |  |  |  |  |  |  |
| Hashad 2016 | case-control | By median | 39 | 51 | 44 | 46 | qPCR | ND1 | HGB | Blood |
|  |  |  |  |  |  |  |  |  |  |  |
| Shen 2016 | case-control | By median | 225 | 168 | 214 | 211 | qPCR | ND1 | HGB | Blood |
